# Response of *Pinus tabuliformis* saplings to continuous autumn fertilization treatments in the mountains of Eastern Liaoning Province, China

**DOI:** 10.7717/peerj.12853

**Published:** 2022-01-28

**Authors:** Yiming Zhang, Jincheng Han, Lijiao Wang, Xin Jing, Yutao Wang, Ping Liu

**Affiliations:** 1 College of Forestry, Shenyang Agricultural University, Shenyang, Liaoning, China; 2 Key Laboratory of Tree Genetics, Breeding and Cultivation in Liaoning Province, China, Shenyang, China; 3 Engineering Technology Research Center of Chinese Pine of National Forestry and Grassland Administration, Shenyang, China

**Keywords:** Autumn fertilization, Growth dynamics, Nutrient change, *Pinus tabuliformis*, Saplings

## Abstract

Autumn fertilization is an important cultivation and management measure used to provide nutrients at the hardening stage during the end of the growing season—bolstering nutrient reserves and promoting additional growth in the following spring. Previous studies mainly focused on short-term or one-time fertilization treatment of container seedlings, and few studies have observed the effects of autumn fertilization of large-area forests over multiple continuous years. The growth dynamics and nutrient changes during autumn in 324 *Pinus tabuliformis* saplings in the temperate zone of China (in the eastern Liaoning mountains) were studied under field conditions with different fertilizer treatments for three consecutive years. The second year of autumn fertilization promoted the growth of tree height and annual leaf length more significantly than that in the first year, the change in diameter at breast height (DBH) was significant. Tree height (TH) in spring increased at a faster rate than in autumn, while DBH stably increased throughout the year. The increase in TH, DBH, and annual leaf length (ALL) under all fertilization treatments was higher than that of the control group, and the decrease in annual branch length (ABL) was higher than that of the control group. High N significantly increased the concentration of new coniferous N (NLN), soil total N (STN), and soil alkali-hydrolyzable N (SAHN) in *P. tabuliformis* saplings. High P significantly increased the concentration of P in annual needles and soil total P (STP), and decreased the concentration of N in new needles. In addition, there is a certain correlation between the N and P concentration in the needles and soil, representing the competition and interactions between plant nutrient demand and soil nutrient supply. The most favorable fertilizer treatment consisted of high N and low P (urea 204 g, calcium superphosphate 104 g), which provide support for the formulation of a reasonable fertilization technology for *P. tabuliformis* in the mountains of Eastern Liaoning Province, China.

## Introduction

Autumn fertilization in trees serves to retain growth capacity, accumulate nutrients for the upcoming year, avoid the effects of nutrient dilution in the later stage of seedling growth, maintain a certain nutrient concentration, as well as for the storage of sufficient nutrients in the seedling. After afforestation, the accumulated nutrients are distributed between the leaves and roots, promoting growth and photosynthesis in the leaves and accelerating the restoration of root vigor. The addition of N can improve the survival rate of seedling afforestation and promote seedling growth (Rikala, Heiskanen & Lahti, 2004). Autumn fertilization has been widely studied in *Pinus ponderosa* (Gleason et al., 1990), *Spruce* (Rikala, Heiskanen & Lahti, 2004), *Black spruce* (Boivin, Miller & Timmer, 2002), and *Eucalyptus globulus* (Fernández et al., 2007)—and is often necessary as young trees continuously accumulate biomass during this season, leading to diluted N storage (Oliet et al., 2011; Wei et al., 2014). Due to climate change and air pollution, with the increasing problem of N deposition, unnecessary fertilization leads to soil and air pollution, in addition to increased production costs. Therefore, a fitted fertilization scheme is necessary to mitigate climate effects and soil pollution. While this has been investigated previously, most of these studies were short-term and implemented one-time fertilization treatment. Further, such studies utilized container seedlings. There have been few studies on autumn fertilization in large woodland areas over period of several years.

Previous studies have found that at low latitudes, the tree growth is limited by P, whereas at mid to high-latitudes, growth is limited primarily by N (Du et al., 2016; Magnani et al., 2007; Jung et al., 2011; Rennenberg et al., 2009; Vitousek et al., 2010). Further, P is the main limiting nutrient in tropical regions with aging soil, while N is usually limited in the temperate regions of the Northern Hemisphere (Reich, Oleksyn & Tilman, 2004). Fertilization experiments using 13 coniferous forests in northern China found that most middle-aged and mature forests exhibited P limitation but not N limitation (Goswami et al., 2018). A large number of studies have shown that the application of N and P fertilizers promotes plant growth and improves productivity, and that the effects of P are particularly better (Broadley et al., 2000; Yang et al., 2014). However, when the total amount of N-deposition caused by fertilizer exceeds the carrying capacity of the ecosystem, a phenomenon known as “N saturation” causes a nutrient imbalance, which leads to reduced rates of photosynthesis (Aber et al., 1989; Sardans, Rivas-Ubach & Peñuelas, 2012; Jiang et al., 2015; Li et al., 2003; Zhang & Liao, 2009). When N deposition increases, P becomes more limited and may become the largest factor limiting ecosystem productivity (Davidson et al., 2007; Cleveland, Townsend & Schmidt, 2002; Walker & Syers, 1976; Zhang et al., 2015). Previous studies have shown that fertilization of young trees in the field is not as effective with very large amounts of fertilizer. Excessive or insufficient fertilization is not conducive to the output of young trees, and cannot achieve the purpose of improving the nutritional status of trees and increasing yield and income, which is consistent with the research on the fertilization effect of most short-rotation tree species (Chen et al., 2020; Hu, 2010; Shu, Yang & Lan, 2009). It has been demonstrated that there is a correlation between N and P concentration and stoichiometry of Larch needles and soil under continuous fertilization, and the correlation between stoichiometry was higher than that of nutrient elements (Niu, 2017). It is known that fertilization has little effect on 1–2-year-old *Cunninghamia lanceolata* (Lamb.) *Hook*. seedlings, but has a great effect on 3-year-old seedlings. Hook forests promote tree height and DBH growth (Chen, 2015b). The DBH difference of short-term fertilization is clear, and with an extension in fertilization time, the DBH difference decreased (Chen et al., 2012).

*Pinus tabuliformis* is a unique representative coniferous tree species found in Northern China. *P. tabuliformis* has a yields good wood, and facilitates soil conservation and wind protection. Further, this species is helpful in forest regeneration and barren mountain afforestation. However, the growth rate is slow and the whole growth and development process can be effectively shortened by improved nurturing techniques. The current research on autumn fertilization of *P. tabuliformis* in China found it can improve the physiological activity of seedling quality, conducive to total nitrogen storage and internal circulation, but the mechanism of nutrient dynamic changes in seedlings is not clear. To shorten the time to maturity of *P. tabuliformis* and improve productivity, the effects of continuous fertilization were studied on *P. tabuliformis* sapling forests over three years in this study. Together, the growth of each index, nutrient concentration of needle leaves, and soil nutrient content—which may indicate whether the treatment can improve seedling afforestation—were assessed. Three hypotheses were tested—whether (1) high-N treatment can increase the nutrient concentration of *P. tabuliformis* saplings trees and soil in temperate regions, (2) high-P treatment can promote the concentration of annual coniferous and total soil P, and (3) soil nutrients promote tree height and DBH.

## Materials and methods

### Study site

**Figure 1:**
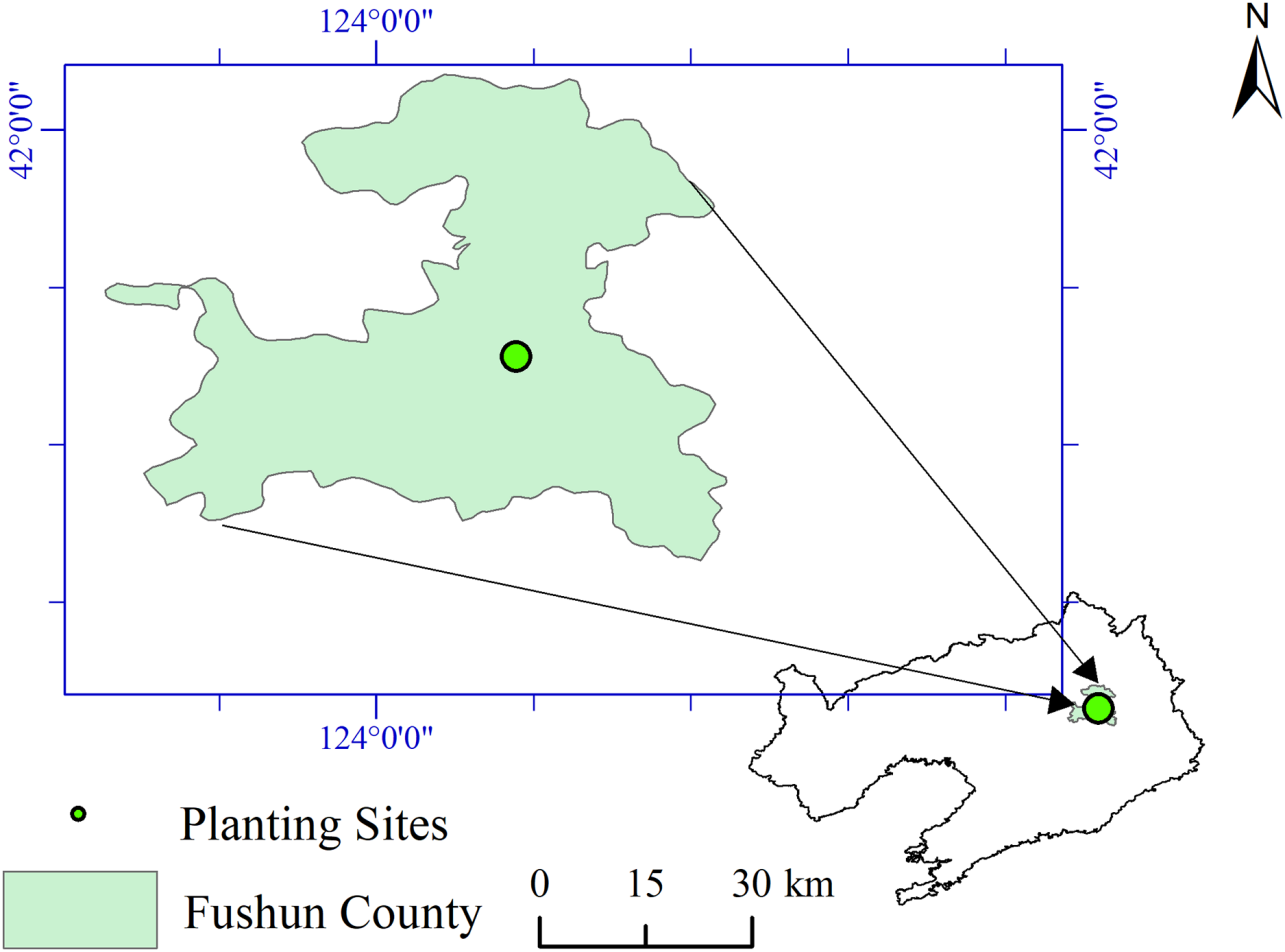
Location of tree planting sites.

The experimental site is located in Magu forest farm, Xiama village, Shangma Town, Fushun County, Liaoning Province, (E123°55′, N41°52′). The site has a continental monsoon climate, with an average altitude of 80 m and a slope of 25° (Fig. 1). On the shady slope, the soil is brown, the thickness of the soil layer is 40 cm, the average annual temperature is 6.6 °C, the average annual precipitation is 804.2 mm, the average annual air relative humidity is 60%, and the frost-free period is 145 days. *Quercus mongolica*, *Juglans mandshurica*, *Rubus crataegifolius*, *Alnus sibirica*, *Lespedeza bicolor*, and *Corylus heterophylla* were the main understory plants.

### Experimental design and N treatment

The experiment was designed as a completely randomized block. The 7-year-old *P. tabuliformis* plantations were set up with 1.5 m row spacing. The concentration of available N and P in the soil were 279.42 mg/kg and 3.74 mg/kg, respectively. Urea and calcium superphosphate were mixed according to the ratio and fertilized by cavity application. A soil pit with a radius of 5 cm was dug at a distance of 15 cm above the roots of the oil pine in each group of 12 trees, and the fertilizer in plastic bottles was poured into the soil pit according to the markings of different treatment groups, and then the soil pit was filled and stepped on with feet. A total of 324 trees were fertilized for three times in October 2018, October 2019 and October 2020. According to the content of soil alkali-hydrolyzable N and soil-available P, three levels (N1: 102g/plant; N2: 204g/plant; P1: 104g/plant; P2: 208g/plant; CK: no fertilizer) were set for each treatment. There were nine plots in total, and each plot contained 12 saplings. An isolation zone, consisting of two lines of *P. tabuliformis* saplings, was set between plots to avoid the edge effect and adjacent treatments. The distribution of fertilization treatments is shown in Table 1. The average initial growth index of *P. tabuliformis* saplings under different fertilization treatments are shown in Table 2.

**Table 1:** Basic information of sample plots and sample trees.

TR/RE	1	2	3	4	5	6	7	8	9
1	N2P2	P2	N1	N2	N1P2	N2P1	P1	N1P1	CK
2	N2	P2	P1	N1P2	N2P2	N1P1	N2P1	N1	CK
3	P2	N1	N2P2	P1	N2	N2P1	N1P1	N1P2	CK

**Table 2:** Initial growth index for *Pinus tabuliformis* saplings.

TR	Average TH/m	Average DBH/cm	Average ABL/cm	Average ALL/cm
N1	3.5	4.8	56.2	10.8
N2	3.7	5.2	57.4	10.7
P1	3.6	4.8	58.1	10.2
P2	3.6	4.7	57.9	10.2
N1P1	3.6	4.9	56.9	11.0
N1P2	3.7	4.9	63.2	10.6
N2P1	3.6	4.8	60.3	10.6
N2P2	3.8	5.0	65.4	10.4
CK	3.5	4.6	56.6	10.0

**Note:**

TH, tree height; DBH, diameter at breast height; ABL, annual branch length; ALL, annual leaf length.

### Sampling and measurements

Sapling height, DBH, annual branch length, and annual leaf length were measured in July and November of 2018–2020. Sapling height and annual branch length were measured using a tower ruler with an accuracy of 0.01 m. DBH was measured using a DBH ruler with an accuracy of 0.1 cm. Annual leaf length was measured using a tape measure with an accuracy of 0.1 cm.

For needle sampling, annual needles were taken from the main branches, and in July of 2019–2020, new needles were taken from the trunk. Twelve *P. tabuliformis* saplings were used for each of the needle measurements. After mixing, needles were placed into envelopes and brought to the lab for chlorosis treatment (105 °C, 60 min) and drying (75 °C, 48 h). After 100 mesh screenings, needles were placed into sealed bags, after which 0.4 g of needles from each treatment were weighed and digested with a solution of sulfuric acid and hydrogen peroxide. Needle total N concentration was measured using indophenol blue colorimetry, and total P concentration using an AA3 flow analyzer.

Soil sampling and measurements were conducted in November of 2018–2020. Soil was collected from the treated fields and brought to the lab for drying and screening. Total soil N and P content were measured using an AA3 flow analyzer. For N content measurements, 0.5 g of air-dried soil was digested with 5 mL of concentrated sulfuric acid and 2 g mixed catalyst (Potassium sulphate copper sulphate selenium was prepared according to the mass ratio of 100:10:1). For total soil P concentration, 0.5 g of air-dried soil (with a 100 mesh sieve) was digested in a furnace with 5 mL of sulfuric acid. Soil alkali-hydrolyzable N content was measured by the alkali diffusion method, and available P in the soil was measured by molybdenum antimony anti-colorimetry (Zhang, 2019).

### Data and statistical analyses

Microsoft Excel 2016 was used for data input and sorting. SPSS 22.0 software was used for data analysis (Stephane et al., 2020). The PWR package of R-studio was used for power analysis (1.3-0 version, UTC, USA), *k* = 9, *n* = 36, *f* = 0.4, sig. Level = 0.05, the calculated power was 99%. Prior to statistical analysis, the original data were tested for normality and homogeneity. For the assessment of statistical significance, three-way (N, P, year) analysis of variance (α = 0.05) was used for complete random design experiments and S-N-K was used as a *post hoc* test to determine differences between groups. Pearson’s correlation analysis was used to analyze the correlation between the nutrient concentrations of needles and soil and growth indexes.

## Results

### The effects of continuous autumn fertilization on the aboveground growth of *P. tabuliformis* saplings grown in the field

**Figure 2:**
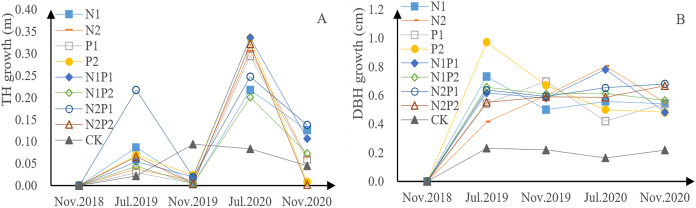
Growth of tree height (A) and diameter at breast height (DBH) (B) in *Pinus tabuliformis* saplings given different fertilizer treatments.

*P. tabuliformis* sapling height, for each fertilizer treatment group and the control, exhibited continuous growth in the form of a bimodal curve (Fig. 2A). The growth rate in 2018 was lower than that in 2019, indicating that continuous fertilization can promote growth. The growth rate of saplings undergoing fertilizer treatment was relatively low from July to November each year, but relatively rapid from November to July of the next year, indicating that there is a period of rapid height increase from November to July. The total growth of tree height in all fertilization treatments was greater than that in the control group (CK), indicating that all fertilizer treatments were able to promote growth in *P. tabuliformis*. Treatment N2P1 yielded the fastest growth, with increases of 0.24 m in 2019 and 0.38 m in 2020. In the first year, N treatment promoted sapling growth and N treatment (N1, N2) had a highly significant effect on tree height (*p* < 0.05), while the effects of P treatment (P1, P2) had a significant effect on tree height (*p* < 0.05; Tables 3 and 4).

**Table 3:** ANOVA results for different aspects of growth, N and P-concentration in the needles and soil content in *P. tabuliformis* saplings.

Variables	N	P	Year	N × P	N × Year	P × Year	N × P × Year
TH	13.396**	5.182*	69.964**	1.269	0.494	0.819	0.521
DBH	150.891**	68.554*	755.426**	110.498**	16.562**	3.491	2.716*
ABL	0.382	1.758	124.571**	1.441	1.084	0.137	0.846
ALL	14.972**	0.140	41.189*	0.848	2.550	1.233	0.565
ACN	27.080**	0.240	5.225*	1.617	0.344	1.221	0.431
ACP	2.547	0.155	0.527	0.896	0.170	0.464	0.244
NLN	66.436**	3.333*	13.743**	2.185	1.871	0.785	1.340
NCP	0.662	0.896	29.319**	2.475	1.685	1.218	1.030
STN	0.785	2.892	27.773**	1.215	0.018	1.667	0.155
STP	0.384	1.210	86.294**	0.943	0.021	0.535	0.672
SAHN	0.356	1.319	11.376**	1.673	0.312	0.070	0.689
SAP	1.795	17.891**	3.213	0.942	0.878	0.279	0.567

**Notes:**

* *p* < 0.05.

** *p*< 0.01.

ACN, annual coniferous N; ACP, annual coniferous P; NLN, new leaf N; NCP, new coniferous P; STN, soil total N; STP, soil total P; SAHN, soil alkali-hydrolyzable N; SAP, soil available P.

**Table 4:** S-N-K results for different aspects of growth, N and P-concentration in the needles and soil content in *P. tabuliformis* saplings.

	Level	1	2	3
TH	N	3.79b	3.86b	4.03a
	P	3.80b	3.80b	3.94a
DBH	N	6.39b	7.09a	7.15a
	P	6.57b	6.92ab	7.13a
ABL	N	38.20a	37.47a	37.01a
	P	37.02a	36.64a	39.03a
ALL	N	11.32b	12.40a	12.64a
	P	12.04a	12.15a	12.16a
ACN	N	1.44c	1.66b	1.80a
	P	1.63a	1.66a	1.63a
ACP	N	0.15a	0.14a	0.14a
	P	0.14a	0.14a	0.14a
NLN	N	1.79c	2.09b	2.54a
	P	2.23a	2.06a	2.13ab
NCP	N	0.19a	0.19a	0.19a
	P	0.18a	0.19a	0.19a
STN	N	0.17a	0.20a	0.19a
	P	0.21a	0.17a	0.18a
STP	N	0.03a	0.03a	0.03a
	P	0.03a	0.02a	0.03a
SAHN	N	228.11a	211.17a	228.61a
	P	244.22a	215.78a	207.89a
SAP	N	6.38a	5.00a	5.56a
	P	3.77b	5.12b	8.05a

**Note:**

Different lowercase letters show significant difference at 0.05 level.

The DBH of the *P. tabuliformis* saplings in each group also exhibited continuous growth (Fig. 2B). The total increase in DBH across all fertilizer treatments was greater than that of the CK, indicating that all fertilizer treatment conditions promoted the DBH. Saplings treated with P2 were the fastest growing, with an increase of 1.64 cm in 2019 and 0.98 cm in 2020. DBH was relatively stable throughout the growth period. There was a highly significant difference with continuous fertilization (*p* < 0.05). The interaction between N and P was significantly different in continuous fertilizer treatments. The effects of P treatment (P1, P2) had a significant effect on DBH (*p* < 0.05; Tables 3 and 4).

**Figure 3:**
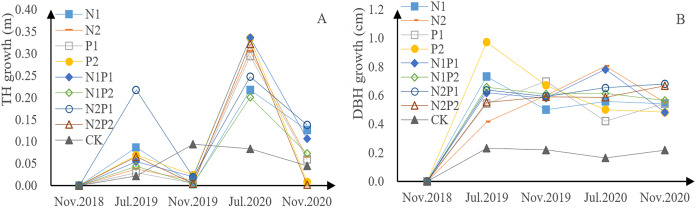
The effects of different fertilizer treatments on annual branch length and leaf growth rate of *Pinus tabuliformis* saplings.

Annual branch length was reduced in all treatment groups (Fig. 3A). The decrease in 2019 was significantly less than that in 2018, indicating that continuous fertilization may promote the decrease in annual branch length. The growth rate of the annual branch length was faster from November to July—likely the inhibition period for this trait. The reduction in annual branch length in each treatment was greater than that in the CK. Treatment N2P1 yielded the largest inhibition in branch growth, with reductions of 19.63 cm in 2019 and 11.26 cm in 2020. However, significant differences between treatments were not found (*p* > 0.05; Tables 3 and 4).

Annual leaf length generally increased in all groups (Fig. 3B), and the increase in 2020 was significantly greater than that of 2019—indicating that continuous fertilization can promote annual leaf length. Overall growth in annual leaf length for all fertilizer treatments was greater than that of the CK, indicating that each fertilizer was able to promote leaf growth. Treatment N2P1 yielded the largest effect, with an increase of 0.59 cm in 2019 and 2.69 cm in 2020. There were highly significant differences in annual leaf length between the N treatments (*p* < 0.05; Tables 3 and 4).

### The effects of continuous autumn fertilization on N and P - concentration in the needles of field-grown *P. tabuliformis*

**Figure 4:**
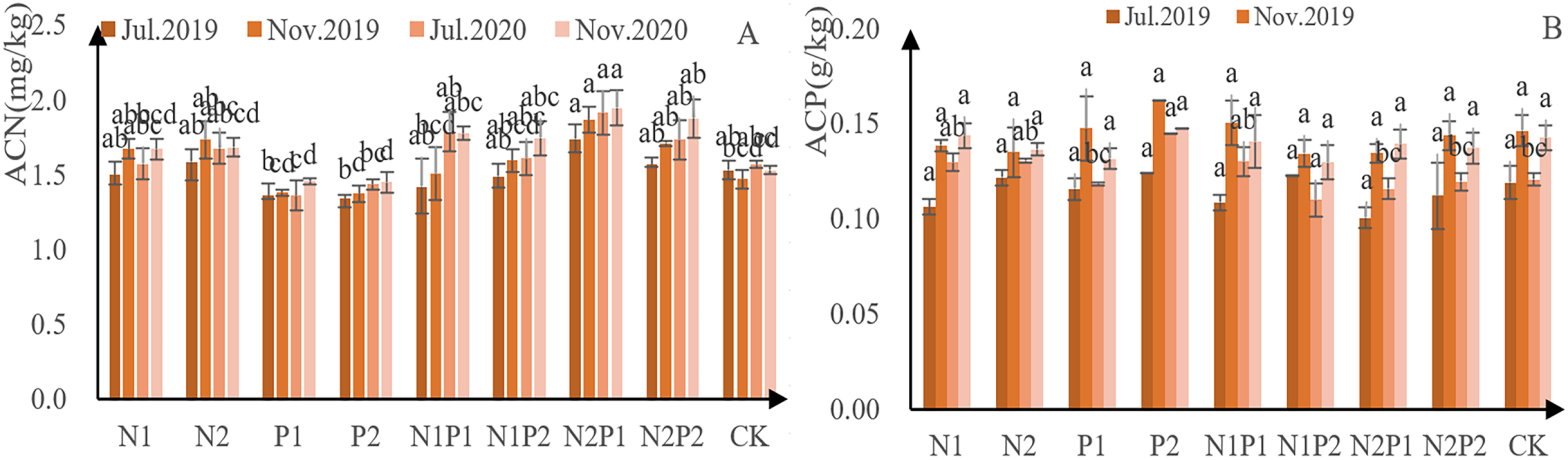
N and P-concentration in the annual needles of *Pinus tabuliformis* saplings given different fertilizer treatments. The different letters over each colored bar represent significant differences (α = 0.05).

With the exception of treatments N1, N2 and P1, N concentration increased continuously in the sapling needles (Fig. 4A). Further, the amount of N absorbed in autumn was higher than that in spring, indicating that autumn fertilization was able to promote the accumulation of N in the needles. Overall, treatment N2P1 had the greatest impact on N concentration—yielding an N concentration of 17.3 g/kg in July 2019, 18.7 g/kg that November, 19.1 g/kg in July 2020, and 19.5 g/kg that November. Compared to the control, needle N concentration decreased with P-only application. The needle N accumulated in the first year of N application, and the effect of fertilizer application in the second year was superimposed, reaching a highly significant level (*p* < 0.05; Tables 3 and 4).

Needle P concentration in the second year was higher than that of the first year, indicating that autumn fertilization had an accumulating effect (Fig. 4B). The P concentration of needles in treatment P2 was the highest—1.24 g/kg in July 2019, 1.62 g/kg in November, 1.45 g/kg in July 2020, and 1.47 g/kg that November. There was no significant difference in P concentration in the annual leaves by continuous application of N fertilization and application of P (*p* > 0.05; Tables 3 and 4).

**Figure 5:**
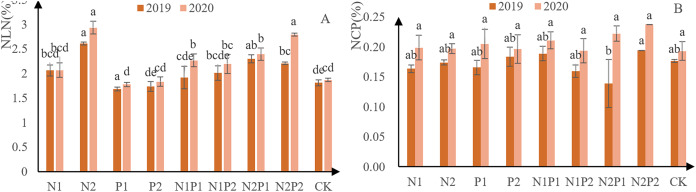
N and P new leaf-concentration measurements from *Pinus tabuliformis* saplings grown under different fertilizer treatments. The different letters over each colored bar represent significant differences (α = 0.05).

The N concentration in new needles from each treatment increased steadily, indicating that all fertilizer treatments promoted N accumulation (Fig. 5A). Except for treatments P1 and P2, N concentration higher than that of the CK in all other all treatments. Overall, treatment N2 performed the best—concentration 26.1 g/kg in 2019 and 29.3 g/kg in 2020. There were highly significant differences in the accumulation of N concentration in new leaves between the different treatments. The effects of P treatment had a significant effect on N concentration in new leaves (*p* < 0.05; Tables 3 and 4).

The N concentration in new needles from each increased steadily, indicating that all treatments were able to promote P (Fig. 5B). Overall, treatment N2P2 performed best, yielding P-concentration of 1.94 g/kg in 2019 and 2.38 g/kg in 2020. There were no significant differences in the accumulation of P in new leaves between the different fertilizer treatments (*p* > 0.05; Tables 3 and 4).

**Figure 6:**
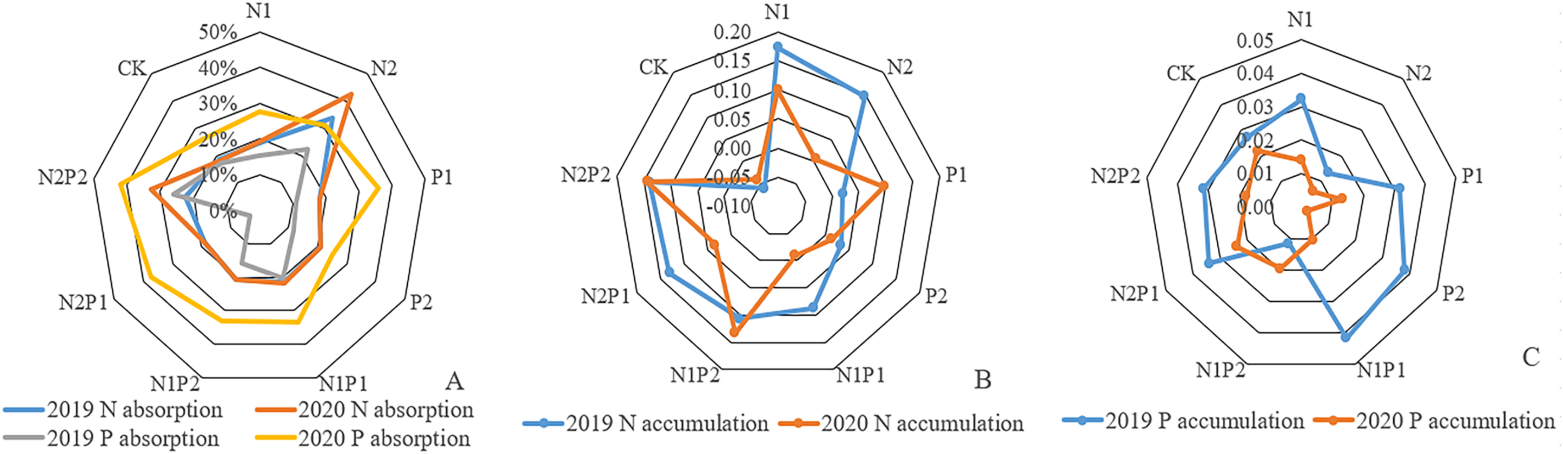
The N-uptake rate, N, and P-accumulation in *Pinus tabuliformis* needles from 2019–2020.

The nutrient absorption rate of total N in needles from all fertilizer treatments, except for N2P1 and P1, was higher than that of the CK. The N absorption rate for treatment N2 was 33.5%, and 22.5% for N2P2. In 2019, N nutrient accumulation reached the highest level, 0.17 g/kg, while other fertilizer treatments remained higher than that of the CK. In 2020, the nutrient uptake rate of total N in the N2 treatment group was the highest, reaching 42.6%, followed by 32.8% in N2P2. Overall, treatment N2P2 yielded the highest accumulation of N, reaching 0.14 g/kg, followed by 0.13 g/kg in N1P2 (Fig. 6).

Except for N1, P1, P2, and N2P1, the nutrient absorption rate for total P in needles in 2019 was higher than that in the CK (Fig. 6). The maximum P absorption rate for treatment N2P2 was 25.9%, followed by 22.3% in N2. In 2019, N1P1 reached the highest level at 0.041 g/kg, while the other fertilizer treatments, except for N2 and N1P2, remained higher than CK. In 2020, the nutrient absorption rates of total P in the needles of all fertilization treatments except P2 were higher than that of CK. The maximum value for N2P2 was 42.2%, followed by 37.2% in N2P1. In 2020, the maximum value of P accumulation in the needles of N2P1 treatment was 0.023 g/kg.

### Effects of continuous autumn fertilization on soil N and P content

**Figure 7:**
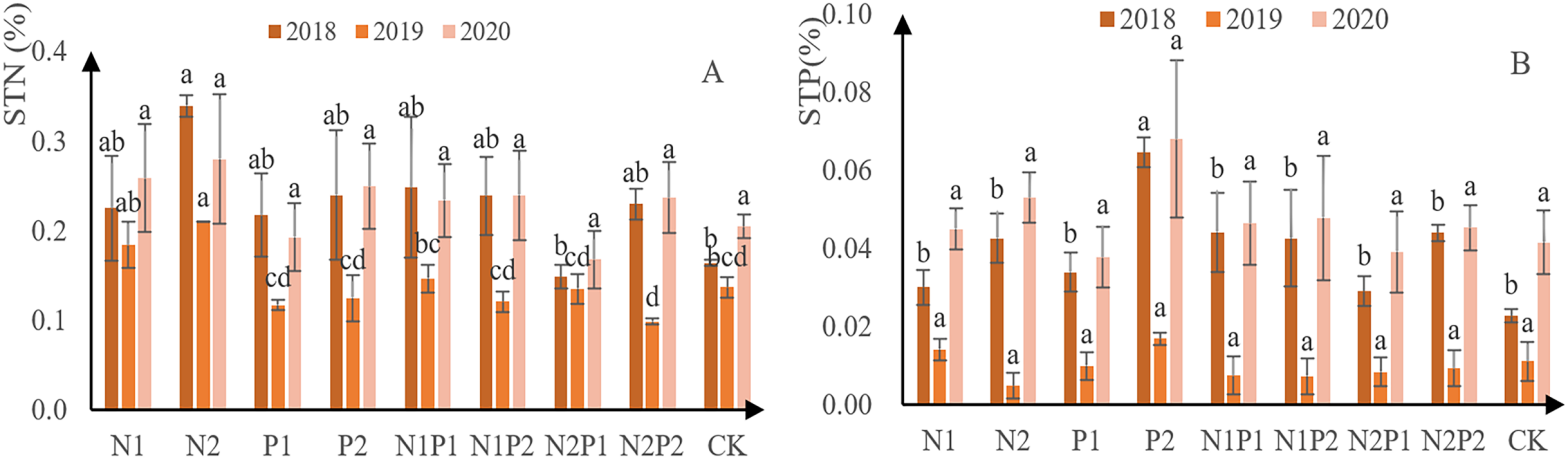
Total soil N and P content at each treatment site. The different letters over each colored bar represent significant differences (α = 0.05).

N concentration was assessed in the soil at the sites where each treatment was conducted. It was found that N content initially decreased and then increased in all treatments, including the CK (Fig. 7A). Overall, N2 was the best treatment for soil N content, yielding 3.4 g/kg in 2018, 2.1 g/kg in 2019, and 2.8 g/kg in 2020. Soil P content followed a similar trend to that for N (Fig. 7B). Overall, P2 was the best fertilizer treatment for soil total P absorption, yielding 0.65 g/kg in 2018, 0.17 g/kg in 2019, and 0.68 g/kg in 2020. There was no significant difference in the soil total N and soil total P contents between the different treatments (*p* > 0.05; Tables 3 and 4).

**Figure 8:**
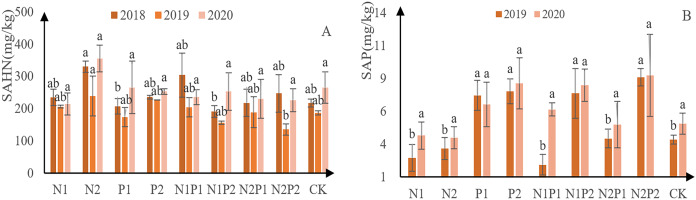
Soil available N and P-contents at each treatment site. The different letters over each colored bar represent significant differences (α = 0.05).

The content of soil alkali-hydrolyzable N at each treatment site decreased initially, followed by an increased (Fig. 8A). Overall, N2 was the best treatment for soil alkali-hydrolyzable N content, yielding 330 mg/kg in 2018, 239 mg/kg in 2019, and 355 mg/kg in 2020. Soil P content generally increasing, with the exception of P1 (Fig. 8B). N2P2 was the best fertilization treatment for soil available P absorption, with 8.6 mg/kg in 2019 and 8.7 mg/kg in 2020. No significant difference was observed between the treatments with soil alkali solution N, and there was a significant difference between the P treatments (*p* < 0.05; Tables 3 and 4).

### Correlation between soil nutrient and needle nutrient and growth index

TH, DBH, ALL, and ABL were significantly positively correlated with STN. There was a significant negative correlation between ABL and STN, STP, and SAHN. New leaf P, TH, DBH, and ALL were significantly positively correlated with STP. New leaf N, TH, and ALL were significantly positively correlated with annual N. TH and ALL were significantly and positively correlated with NLN. There was a significant positive correlation between the NLP and ALL. There was a significant negative correlation between ABL and NLL. DBH and ALL were significantly and positively correlated with TH. There was a significant positive correlation between ALL and DBH. There was a significant negative correlation between ABL and DBH. There was a significant positive correlation between NLN, NLP, and STN. There was a significant positive correlation between tree height, DBH, and SAHN. DBH was positively correlated with N and P in new leaves (Table 5).

**Table 5:** Correlation coefficients for soil to needle nutrient-contents and growth indexes.

Variables	STN	STP	SAHN	ACN	ACP	NLN	NCP	TH	DBH	ABL
STP	0.823**	1								
SAHN	0.772**	0.691**	1							
ACP	−0.346	−0.156	−0.196	−0.544*	1					
NLN	0.496*	0.225	0.396	0.797**	−0.503*	1				
NCP	0.513*	0.681**	0.414	0.400	−0.006	0.314	1			
TH	0.623**	0.726**	0.490*	0.626**	−0.368	0.631**	0.747**	1		
DBH	0.683**	0.676**	0.518*	0.444	−0.362	0.541*	0.543*	0.855**	1	
ABL	−0.0775**	−0.871**	−0.622**	−0.359	0.385	−0.318	−0.599**	−0.733**	−0.634**	1
ALL	0.647**	0.602**	0.417	0.677**	−0.467	0.670**	0.619**	0.846**	0.859**	−0.739**

**Notes:**

** Significant at the 0.01 level.

* Significant at the 0.05 level.

## Discussion

### Response of the aboveground growth of *P. tabuliformis* saplings to continuous fertilization

Fertilization is beneficial to the growth of *P. tabuliformis* saplings, particularly at the fast-growing stage. Fertilization greatly increases the nutrient demand for the growth and development the sapling, significantly promoting the growth of the forest, which is consistent with results described by Lu et al. (2019). Continuous fertilization during autumn can promote the growth of DBH and height of *P. tabuliformis* saplings, which is consistent with one of our earlier hypotheses (Liang et al., 2019). In subtropical areas, high N and low P is known to increase height in *P. tabuliformis*, while high P can promote DBH growth. However, disagree with the results of this study. It may be that compound fertilizer can meet growth needs, and that nutrients have different effects on DBH and height (Chen et al., 2020; Chen et al., 2021). In the mountains of Eastern Liaoning Province, fertilizer treatment had no significant effect on annual branch length or leaf length—consistent with the results of Zhang (2019). In contrast, Ma et al. (2018) demonstrated that treatment with compound fertilizer significantly increased height in 2-year-old *Betula alnoides* but had no significant effect on DBH. In the second year after fertilization, fertilization treatment significantly promoted tree height, which may be due to the inconsistent response of DBH and height growth of different tree species to fertilizer and its species combination. Further research is required on different tree species. It has been shown that fertilization during autumn can promote the growth and nutrient accumulation of seeding beyond that observed in the spring. At the same time, seeding growth was promoted more significantly in the second year after autumn fertilization—similar to the effects of field fertilization (Sun, 2020).

### N and P-concentration in the needles of *P. tabuliformis* saplings treated with continuous fertilization

The P concentration of annual needles first increased and then decreased, which is consistent with previous work (Bai, 2016). It is likely that in the early stages of needle development, the cells are in a state of rapid division, requiring an abundance of resources for protein and nucleic acid production. Thus, the P concentration in needle leaves is higher. During growth and development, the leaves grow rapidly, and their biomass increases while nutrient are gradually diluted. This process exhibits obvious seasonal changes as well as differences at different life cycle stages in the plant (Kerkhoff et al., 2005). This study further confirmed that proper application of N fertilizer can enhance the absorption and accumulation of N in plants, which is similar to the existing research results, and can help explain why the average N concentration in needles under multi-year fertilization treatments (N1, N2, N1P1, N2P2, N1P2, and N2P1) was significantly higher than that of treatment P or control groups (Jiang et al., 2015; Wu, Bao & Wu, 2008). The results show that P addition can increase P concentration in annual needles, which is consistent with our second hypothesis (Li et al., 2016; Wang et al., 2014). Consistent with the results of this study, N application alone can significantly increase N concentration in needles, while the P concentration in needles decreases (Amponsah et al., 2005; Chen, 2015a). A large number of substances in plants contain N, and compared with any other nutrient element in the plant, the demand for N is quite high (Cruz et al., 2003; Reich, Oleksyn & Tilman, 2004). Some studies have shown that due to the close relationship between N and P in plants, these elements can promote absorption, utilization, and transformation of each other.

Overall, our findings demonstrated that P concentration was not significantly affected by continuous N addition in the annual conifer *P. tabuliformis*. Further, we showed that P concentration was higher in the plants themselves, and that N was not the limiting factor for P concentration in the needles of *P. tabuliformis*. These results further confirmed that the trees were restricted by N and P at different sites. There are significant differences between different species. It can be concluded from the above analysis that the combined application of N and P will increase each other’s consumption. Although the application of N (P) can increase the content of N (P) in plants, the lack of P (N), another important component, still limits the absorption and transformation of nutrients. Therefore, increasing the content of N and P at the same time is more beneficial to the coniferous content of *P. tabuliformis* saplings and has a promoting effect on tree growth and forest productivity (Jiang et al., 2018; Li et al., 2018).

### Response of soil N and P-content to continuous fertilization

Soil total N, P, and soil alkali-hydrolyzable N all showed a decreasing trend at first followed by an increase, which could be due to several reasons. July is the most vigorous growth period for these plants, meaning they must absorb more nutrients from the soil to meet the growth and development requirements. At this time, the forest land temperature is higher, and the soil N mineralization rate is high, so it can be reduced to a certain extent (Hu & Sun, 2020). According to previous studies, the absorption of N and P was higher in the first year after planting. After this age, 30% of the annual N and P demand can be met through internal migration, and the nutrients stored in leaves have a dilution effect, reducing the nutrient concentration in leaves (Melo et al., 2015). In contrary to the results of this study, Pei (2015) indicated that exogenous N addition affected the return rate of litter nutrients, increased the decomposition rate of soil organic matter to a certain extent, and increased the total P content of soil. A possible reason for this discrepancy is that the single forest land and long-term application of N fertilizer will easily lead to soil acidification and affect the decomposition of soil organic matter. The available soil P content is known to increase significantly after single-application of P fertilizer (Wei et al., 2011). N application alone reduced the content of soil available P, which may be because N application increases the content of soil available N, increases the absorption of N by plants, promotes the absorption of available P by plants, and reduces the content of soil-available P.

### Correlations between needle and soil nutrient content with growth index

The addition of N and P in soil can affect the physiological metabolism of trees. Many related studies have reported that there is an inevitable link between leaf and soil nutrients (Bai et al., 2016; Zeng et al., 2015; Zhao et al., 2016). The leaf is the most active organ in plant physiological metabolism, and is considered to be the most sensitive part to the change in soil nutrient supply. The change in leaf nutrient concentration is mainly affected by the phenological period and soil nutrient supply (Kim et al., 2015; Liu et al., 2015). In the case of insufficient nutrient supply, there was a strong correlation between the leaf nutrient concentration of trees, soil nutrient supply level, and tree growth. However, in the case of rich soil nutrient content, there was no significant correlation between leaf nutrient concentration and soil nutrient supply (Zhao et al., 2010). In this study, N addition led to an increase in soil total N, soil alkali-hydrolyzable N, and coniferous N—indicating that the supply of soil nutrients was insufficient. The branches and trunks of trees are important organs for nutrient storage, and their nutrient content is a direct response to the soil nutrient supply. Fertilization not only affects the growth of trees but also affects the forest nutrient cycle, which is consistent with our third hypothesis.

Relevant studies have shown that there is a mutual promotional relationship between soil total and new leaf -P (Niu, 2017). This relationship may be related to the fact that the P content in the soil of an ecosystem is lower than the global average level, which leads to a lower P concentration in plant leaves and may cause P limitation. DBH in *P. tabuliformis* saplings was significantly positively correlated with soil total P, soil available N, soil total N, and soil available P, indicating that P and N in the soil were the main elements promoting DBH growth. Annual branch length was significantly negatively correlated with soil total N, soil total P, and soil alkali-hydrolyzable N, which indicated that the growth of annual branch length was limited by soil nutrients, and the slope aspect also affected the annual branch length (Che, 2019). The results showed that the N concentration in leaves was positively correlated with tree height and DBH, and P concentration was positively correlated with tree height and DBH, which was consistent with previous results. Therefore, the management of fertilization and tending should be further strengthened according to the characteristics of leaf nutrient absorption and accumulation (Lin et al., 2020). With the passage of time, the total amount of N deposition continued to increase and eventually exceeded the sustainable range of the forest ecosystem, which would have an impact on the N cycle, soil characteristics, and biomass of the ecosystem (Chen, 2015a; Lin & Xue, 2020; Lu, 2015; Wang et al., 2020).

## Conclusions

Autumn fertilization can alleviate the nutrient dilution effect in the late growth stage of seedlings, maintain a certain nutrient concentration, store sufficient nutrients in seedlings, improve the survival rate of seedling afforestation, and promote biomass and nutrient accumulation more than spring fertilization and summer fertilization. Continuous fertilization during autumn is able to promote the growth of DBH and tree height of *P. tabuliformis* saplings during the fast-growing period. Continuous N addition increased N concentration in new coniferous N, soil total N, and SAHN, while the P concentration in needles decreased without significant effect. Autumn fertilization not only affects stand growth, but also affects needle and soil nutrient cycling processes, responding to the competition and interactions between plant nutrient demand and soil nutrient supply. This study only investigated the effects of different fertilization treatments on the growth of *P. tabuliformis* saplings. In the future, it would be helpful to study the comprehensive effects of other factors on the growth of *P. tabuliformis*.

## Supplemental Information

10.7717/peerj.12853/supp-1 Supplemental Information 1 Raw data.

## Additional Information and Declarations

### Data Availability

The following information was supplied regarding data availability: The raw data are available in the Supplemental File.
